# Biomolecular condensates as arbiters of biochemical reactions inside the nucleus

**DOI:** 10.1038/s42003-020-01517-9

**Published:** 2020-12-15

**Authors:** Guillaume Laflamme, Karim Mekhail

**Affiliations:** 1 Department of Laboratory Medicine and Pathobiology, Faculty of Medicine, University of Toronto, MaRS Centre West Tower, 661 University Avenue, M5G 1M1, Toronto, ON Canada; 2 Canada Research Chairs Program, Faculty of Medicine, University of Toronto, Medical Sciences Building, 1 King’s College Circle, M5S 1A8, Toronto, ON Canada

**Keywords:** Nuclear organization, Epigenetics, DNA damage and repair, Chromatin, Gene expression

## Abstract

Liquid-liquid phase separation (LLPS) has emerged as a central player in the assembly of membraneless compartments termed biomolecular condensates. These compartments are dynamic structures that can condense or dissolve under specific conditions to regulate molecular functions. Such properties allow biomolecular condensates to rapidly respond to changing endogenous or environmental conditions. Here, we review emerging roles for LLPS within the nuclear space, with a specific emphasis on genome organization, expression and repair. Our review highlights the emerging notion that biomolecular condensates regulate the sequential engagement of molecules in multistep biological processes.

## Abstract (web summary)

Laflamme and Mekhail discuss emerging nuclear roles for LLPS in genome organization, gene expression and DNA repair, highlighting the emerging notion that biomolecular condensates regulate the sequential engagement of molecules in multistep biological processes.

## Introduction

Eukaryotic cells are composed of a variety of organelles, which are typically enclosed by a membrane composed of a lipid bilayer. The main function of these membrane-bound organelles is to organize and compartmentalize the cell and to favour certain types of molecular reactions. This is in part achieved by increasing the concentrations of specific factors and by sequestering and storing other molecules. One such organelle is the nucleus, which is delimited by a double lipid bilayer called the nuclear envelope. The transport of molecules from the cytoplasm to the nucleus occurs through nuclear pore complexes that are embedded within the nuclear envelope.

In addition to membrane-bound organelles, cells contain a variety of membraneless compartments. Increasing evidence suggests that LLPS underlies the formation of such biomolecular condensates^1–3^. Phase separation is defined as the process by which macromolecules condense into a liquid-like dense phase, demixing from a diluted surrounding environment^2,4^. LLPS allows for the enrichment of specific factors while excluding others from the condensates, thereby creating a unique environment that either favours or restricts certain biochemical reactions^5^. LLPS drives the formation of multiple membraneless compartments, including but not limited to P granules, the nucleolus, facultative heterochromatin, transcriptional complexes and DNA repair centres^6–14^. These phase-separated compartments are dynamic, and the processes mediating their formation and dissolution are tightly regulated. Indeed, aberrant transition in phase separation from a liquid-like state to a more solid-like state has been linked to various human illnesses, including neurodegenerative diseases and cancer (reviewed elsewhere^4,15–18^).

In this review, we provide an overview of the criteria used to define LLPS and the mechanisms regulating this process. We also discuss recent LLPS literature with a focus on genome organization, expression and repair. Overall, we argue for a complex interconnection between different phase-separated intranuclear compartments.

## LLPS characteristics and criteria

Membraneless organelles that form through the process of LLPS are composed of macromolecules, such as proteins and nucleic acids, which self-organize into a dynamic network of multivalent interactions. The formation of this dynamic network relies on forces promoted by weak intra- and intermolecular interactions including a combination of hydrophobic, electrostatic, cation-pi and pi-pi contacts^19–21^. These weak interactions allow for the rapid internal reorganization of the biomolecular condensates. The specific interactions between macromolecules within the condensates enable the formation of distinct and specialized compartments^5^.

Multivalency, or the presence of multiple interacting domains or elements in macromolecules, is also a critical factor mediating LLPS^2,22,23^. This multivalency can be achieved through the presence of repeated structured domains within proteins^1,21^. In addition, due to its capacity to form scaffolds and connect its multiple binding partners, RNA is an especially central player in phase separation^24–27^. Multivalency can also be driven by the intrinsically disordered regions (IDRs) of proteins^13,28,29^. IDRs do not adopt a stable three-dimensional structure, allowing IDR-containing proteins to serve as scaffolds that interact with short and flexible interacting motifs^30,31^. Of note, IDR mutations are frequently observed in diseases associated with LLPS dysregulation. These observations highlight the importance of the precise and dynamic regulation of biomolecular condensates. This regulation can be achieved, at least in part, via post-translational modifications (PTMs) of condensate components, which can greatly modify the charge and the conformational space of IDRs^32–35^. However, it is also important to note that IDRs are not always drivers of LLPS, and that IDRs can even inhibit LLPS in specific contexts^29,36^.

The notion that membraneless cellular organelles can form through LLPS is not a new concept. Indeed, in 1946, after studying the effect of temperature variation on the size of the nucleolus, Lars Enrenberg stated that “the nucleolus is a separated phase out of a saturated solution”^37^. More recently, the nucleolus, along with other major nuclear compartments, has often served as a model to study LLPS processes within the nucleus^7,38,39^. These studies have highlighted a number of specific criteria for defining LLPS both in vitro and in vivo (reviewed elsewhere^1,3,40,41^). First, due to surface tension, the shape of a biomolecular condensate is most often spherical^40^. Importantly, this spherical shape can be deformed when a physical force is applied or when biomolecular condensates flatten against a surface, a phenomenon known as wetting^13^. Second, biomolecular condensates often fuse upon touching before relaxing back into a spherical shape^40^. Third, biomolecular condensates contain highly dynamic molecules that exchange both within the condensate and with the surrounding environment. This rapid rearrangement of molecules in biomolecular condensates can be assessed by fluorescence recovery after photobleaching (FRAP)^1^. Fourth, biomolecular condensates assemble only after its components reach a saturation concentration (C_sat_)^40^. Moreover, the size of the condensate is proportional to the concentrations of its components. Fifth, the movement of components across the phase separation boundary is energetically unfavourable due to the presence of cohesive interactions within the condensate. This results in a decreased diffusion rate for molecules moving across a phase boundary^3^. Finally, treatment with the aliphatic alcohol 1,6-hexanediol can be used to assess the involvement of weak hydrophobic interactions during the formation of biomolecular condensates^13^.

Next, we will focus our discussion on three types of membraneless compartments suggested to form through LLPS inside of the nucleus. We will provide evidence for and against the involvement of phase separation in the formation of these compartments, as well as the key factors implicated in the dynamic regulation of these structures.

## LLPS in heterochromatin and genome organization

In eukaryotic cells, DNA is packaged and wrapped around an octameric complex of histone proteins. These structures, called nucleosomes, represent the first level of DNA compaction and are often referred to as “beads on a string”^42^. These nucleosomes are then further organized and compacted to various degrees to form chromatin fibres. Heterochromatin, or silent chromatin, is critical for eukaryotic genome regulation and organization. For instance, heterochromatin formation regulates the activity of mobile genetic elements and supports the sequestration and stabilization of repetitive DNA sequences^43–47^. A key feature of heterochromatin is histone H3 trimethylated on lysine 9 (H3K9Me3), which is deposited by the histone methyltransferase Suv39h in mammals^48^. The spreading of this epigenetic mark along chromatin allows for the binding of the highly conserved heterochromatin protein 1 (HP1)^49^. HP1 proteins associate with H3K9Me3 via their N-terminal chromodomain and dimerize through their C-terminal chromoshadow domain^50–52^. Together, HP1 proteins form a platform and allow the recruitment of additional heterochromatin factors that mediate chromatin compaction and transcriptional silencing.

**Fig. 1:**
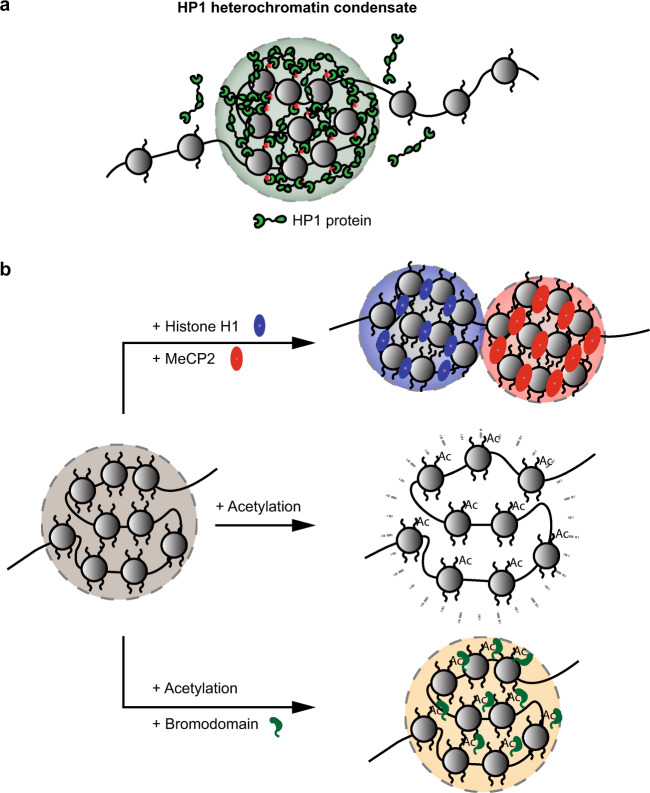
LLPS in heterochromatin and genome organization. **a** HP1 proteins associate with H3K9Me3 marks (red), oligomerize, and mediate the LLPS of heterochromatin. **b** Factors controlling the LLPS of nucleosome arrays. Biomolecular condensates formed by chromatin are modulated by several factors. Addition of the linker histone H1 (blue) and MeCP2 (red) proteins results in the formation of distinct and immiscible condensates. Acetylation of histone tails leads to the dissolution of the condensate, resulting in a more dispersed arrangement of the nucleosomes. Chromatin condensates can be reassembled upon the addition of bromodomain-containing proteins (green).

Recently, HP1-mediated heterochromatin formation was suggested to involve LLPS (Fig. 1a)^8,9,53^. Indeed, HP1 proteins purified from *Drosophila melanogaster*, *Homo sapiens* and *Schizosaccharomyces pombe* all undergo phase separation in vitro. Specifically, human HP1α proteins require either the phosphorylation of their N-terminus or the presence of DNA for phase separation in vitro^9^. Similarly, liquid droplet formation by the *S. pombe* HP1 protein Swi6 also requires the presence of either DNA or nucleosomes^53^. Most in vivo experimental observations of HP1 are also consistent with the process of LLPS. For example, *Drosophila* HP1a exhibits liquid-like properties in early embryos, as GFP-HP1a foci are spherical and frequently fuse. More importantly, reductions in the diffusion rate of HP1a at the heterochromatin–euchromatin border reveal the presence of a phase boundary^8^. Mechanistically, the binding of the *S. pombe* HP1 protein Swi6 to nucleosomes was shown to result in dynamic conformational changes and destabilization of the nucleosome. Such changes result in the exposure of buried nucleosomal regions, providing additional opportunities for multivalent interactions between nucleosomes and promoting LLPS^53^.

However, not all observations of heterochromatin compartments are compatible with HP1-driven phase separation. First, FRAP experiments indicated the presence of an immobilized fraction of *Drosophila* HP1a proteins in the late stages of developing embryos^8^. The immobilization of HP1 on chromatin suggests that the dynamic organization of heterochromatin through LLPS could reorganize into a more static structure during development or under certain conditions. Furthermore, studies in mouse embryonic fibroblasts indicated that HP1α organizes heterochromatin into a collapsed polymer globule without LLPS hallmarks^54^. Future work should aim to understand how heterochromatin-related phase separation may differ across developmental stages and organisms. It will be important to identify the factors and mechanisms driving the physiological transition of heterochromatin from a liquid-like state to other states. Such information could also shed light on pathological phase transitions observed in the context of cancer or neurological disorders.

Beyond heterochromatin, a more general capacity of chromatin to undergo LLPS is emerging. Purification of recombinant dodecameric nucleosome arrays incubated at physiological salt concentrations revealed that LLPS is in fact an intrinsic property of chromatin and can be modulated by several factors (Fig. 1b)^55^. The addition of linker histone H1 proteins resulted in denser and less dynamic chromatin droplets, whereas histone acetylation triggered the dissolution of these droplets. The addition of bromodomain-containing proteins such as BRD4, which binds to histone tails with acetylated lysines, restored the LLPS of acetylated chromatin^56^. In addition, distinct chromatin phases that are composed of acetylated and non-acetylated chromatin can coexist without coalescing^55^. Other factors, such as methyl-CpG-binding protein 2 (MeCP2), which selectively binds methylated CpG dinucleotides, can also induce LLPS of nucleosomal arrays. Of interest, MeCP2 competes with linker histone H1 and forms distinct heterochromatin condensates that coexist but do not mix (Fig. 1b)^57^. The LLPS of nucleosome arrays likely acts on a shorter length scale within chromosomes. This might in turn contribute to the dynamic compartmentalization of the genome via the formation of relatively granular chromatin subdomains with different degrees of compaction. Overall, these studies indicate that the intrinsic capacity of chromatin in general, or heterochromatin in particular, to undergo LLPS can be actively modulated by histone or DNA modifications as well as chromatin binding factors. Importantly, chromatin regulation constitutes only one approach that LLPS can employ to control gene expression.

## LLPS in gene expression

Eukaryotic gene expression is regulated by an array of transcription factors and coactivators that bind to and connect promoters with enhancers. Transcription factors enable the recruitment of RNA polymerase II (RNA Pol II) to promoter regions to activate transcription^58^. The clustering of multiple enhancers in three-dimensional space can form super-enhancers where the transcription machinery components become highly enriched. Such super-enhancers can efficiently boost and/or coordinate gene expression at proximal genes and play a pivotal role in determining cell identity during development^59,60^.

**Fig. 2:**
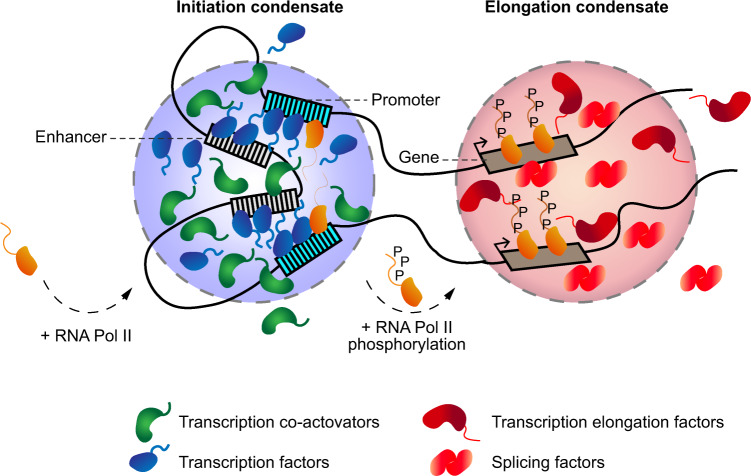
LLPS in transcriptional regulation. Biomolecular condensates composed of transcription factors and coactivators at super-enhancers and promoter sequences recruit RNA Pol II. Phosphorylation of RNA Pol II mediates its transition from the initiation condensate to the elongation condensate, which is composed of transcriptional elongation and splicing factors.

The function of super-enhancers may also be shaped by LLPS^10,61^. Consistent with this notion, transcription factors and coactivators commonly contain IDRs that can compartmentalize and concentrate factors necessary for transcription (Fig. 2)^62–68^. For example, endogenous levels of the transcriptional coactivators BRD4 and a component of the Mediator complex (MED1) are enriched at super-enhancers^62,63^. The structures formed by these coactivators show liquid-like properties in cells. These properties include frequent fusion events, dynamic internal rearrangements of the components as evidenced by FRAP and dissolution of the foci upon 1,6-hexanediol treatment^62,64^. In vitro, many purified transcription factors and coactivators containing IDRs organize into phase-separated droplets that are capable of mixing with MED1 droplets^63^. Recently, the ability of BRD4 to undergo LLPS was shown to be dependent on the expression of specific isoforms and on phosphorylation^68^.

Additional factors, including members of the FUS, EWS and TAF15 (FET) family of RNA and DNA binding proteins, contain IDRs and undergo LLPS in vitro^65,69,70^. Proteins of the FET family also exhibit LLPS properties in vivo when tethered to artificial *LacO* arrays or endogenous microsatellite repeats. FET proteins form spherical and highly dynamic compartments that are capable of selectively enriching specific factors, including RNA Pol II. Interestingly, the concentration of FET proteins at the *LacO* site significantly exceeded the number of binding sites, suggesting the existence of cooperative binding^65^. Recent evidence also suggests that transcriptional control of the Hippo pathway, which is involved in various aspects of cell proliferation and differentiation^71^, is regulated by LLPS^66,67^. Under osmotic stress, YAP and TAZ transcriptional coactivators exhibit liquid-like properties in vitro and in vivo. Similar to other transcriptional coactivators, YAP and TAZ nuclear condensates co-localize with many other transcription mediators, including BRD4, MED1 and RNA Pol II^66,67^. Taken together, these studies suggest that LLPS at super-enhancers enables the recruitment and concentration of transcription regulators that interact through their IDRs to enrich RNA Pol II and control gene expression.

In addition to concentrating the factors involved in transcription initiation, recent evidence also suggests that LLPS can promote and coordinate the process of transcription elongation (Fig. 2)^58^. In this model, phosphorylation of the C-terminal domain (CTD) of RNA Pol II by CDK7 and CDK9 kinases enables the transition of RNA Pol II from initiation condensates to elongation condensates^72–74^. These elongation condensates are at least in part composed of the positive transcription elongation factor b (P-TEFb) and various splicing factors^73,74^. The P-TEFb component cyclin T1 contains an IDR and shows LLPS properties in cells, including fusion events and sensitivity to 1,6-hexanediol^73^. Specifically, in vitro, the phosphorylated form of the RNA Pol II CTD was shown to preferentially incorporate into SRSF1- and SRSF2-containing splicing factor condensates compared to mediator condensates^74^. Together, these results highlight the ability of PTMs to rapidly modulate the composition and shuttling of factors between adjacent, yet distinct, biomolecular condensates.

A key consideration when assessing the capacity of macromolecules to undergo LLPS in vivo is the level of expression of the factor of interest. As already stated, biological condensates assemble only after reaching C_sat_, making them extremely sensitive to changes in protein concentration^40^. Although exogenous expression of molecular players can reveal their capacity to assemble into biomolecular condensates, caution should be used when employing such an approach. For example, as mentioned above, clear evidence for LLPS was observed upon gross overexpression of FET proteins (FUS and TAF15). However, even if endogenous levels of FET proteins lead to the formation of dynamic hubs that show selectivity for binding partners and RNA Pol II, LLPS features were not observed under these conditions^65^. This highlights the importance of studying LLPS processes using different systems under physiologically relevant expression levels, as performed in different studies^13,62,64,74–76^.

## LLPS and the spatiotemporal regulation of DNA repair

**Fig. 3:**
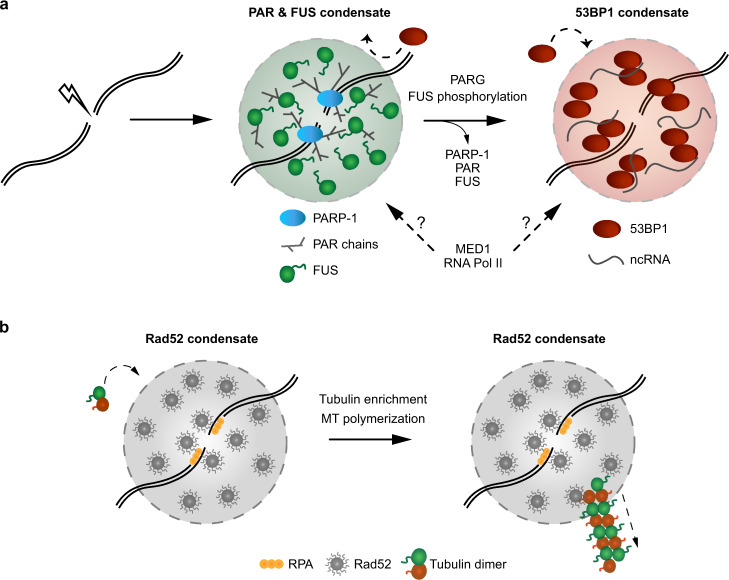
LLPS in the compartmentalization of damaged DNA. **a** Mammalian PARP-1 is an early responder at DNA damage sites and synthetizes long negatively charged PAR chains that interact with positively charged RGG motifs present on FUS proteins. LLPS of FUS allows the compartmentalization of the DNA damage site and the exclusion of specific factors, such as 53BP1. Upon PARG activation and FUS phosphorylation, the FUS condensate disassembles and allows for the enrichment and subsequent LLPS of 53BP1. This phenomenon is mediated, at least in part, by the oligomerization domain of 53BP1. The enrichment of the mediator component MED1 and RNA Pol II in either compartment remains to be determined. **b** Budding yeast Rad52 forms biomolecular condensates at sites of DNA damage. The condensates can concentrate tubulin and drive the polymerization of microtubule filaments. The filaments help mobilize damaged DNA inside of the nucleus to promote repair.

Cellular health and longevity are continuously threatened by various sources of DNA damage. Therefore, cells have evolved several complex DNA repair pathways. Mounting evidence suggests that different DNA repair pathways rely on LLPS for efficient repair (Fig. 3a)^12,13,70,75–77^. Among the early responders at sites of DNA damage are members of the ADP-ribosyltransferase (PARP) family^78^. At the site of DNA damage, PARP-1 synthetizes long negatively charged poly(ADP-ribose) (PAR) chains that are subsequently removed by the PAR glycohydrolase PARG^79^. It has been proposed that PAR could function as a molecular seed to promote the assembly and LLPS of IDR-containing proteins at the site of DNA damage^12^. After irradiation-induced DNA damage, members of the FET family rapidly accumulate at DNA damage sites in a PAR-dependent manner^80,81^. This accumulation is promoted by electrostatic interactions between negatively charged PAR chains and unstructured positively charged arginine-glycine-glycine (RGG) motifs present on FUS and other FET proteins^82^. The structural flexibility of RGG repeats promotes their multivalent electrostatic interactions with PAR chains to promote LLPS^83^. Indeed, the accumulation of FET proteins at sites of laser microirradiation is associated with changes in light diffraction, suggesting that the refractive index and mass density at the site of damage differ from those of its surrounding environment upon FET recruitment^12^. Moreover, FET proteins at the site of DNA damage undergo dynamic exchange with the surrounding nucleoplasm, as revealed by FRAP analysis^70^. In vitro, activated PARP-1 and FUS lead to the formation of large compartments that are specifically enriched with damaged DNA^77^. The phase separation properties of FUS may be tightly regulated during the DNA damage response^84^. For instance, DNA-PK-dependent phosphorylation of FUS prevents its phase separation in vitro^33,34^. On the other hand, such DNA-PK-driven phosphorylation of FUS can promote its localization to the cytoplasm and aggregation into fibrils^84,85^. These seemingly contradictory effects of DNA-PK-dependent phosphorylation of FUS on its phase separation behaviour in the nucleus and cytoplasm may reflect, at least in part, the different interactomes of FUS in these subcellular compartments. The transient compartmentalization of damaged DNA by PAR and FUS may contribute to the spatiotemporal regulation and sequential recruitment of DNA repair proteins. For example, the genome caretaker protein 53BP1 appears to be excluded from these PAR/FUS compartments and can only access sites of DNA damage upon PAR and FUS removal^12^. 53BP1 foci at the sites of DNA damage also exhibit liquid-like properties, including frequent fusion and fission events and sensitivity to 1,6-hexanediol^75,76^. Of note, the ability of 53BP1 to undergo LLPS depends on its oligomerization domain instead of its unstructured N-terminal domain^75^. Importantly, this oligomerization domain is also responsible for the multimerization and accumulation of 53BP1 at the site of DNA damage^86^. In addition, the recruitment and dynamic behaviour of 53BP1 are modulated by the presence of long non-coding RNAs transcribed from the site of DNA damage^76,87–89^. This local transcription is promoted by MRN-dependent recruitment of the Mediator protein MED1, transcription factors of the preinitiation complex, and RNA Pol II at DNA double strand breaks (DSBs)^76^. In light of the capacity of MED1 and RNA Pol II to undergo LLPS at super-enhancers, it is possible that these factors also directly promote the assembly of molecular condensates at DNA damage sites.

The role of phase separation in DNA repair appears to be evolutionarily conserved. Work performed with *Saccharomyces cerevisiae* revealed that the DNA repair and recombination protein Rad52 exhibits liquid-like behaviour in vitro and in vivo^13^. Surprisingly, sequential fusion of smaller Rad52 droplets inside of the nucleus results in a large liquid-like Rad52 droplet that becomes enriched in tubulin before projecting microtubule filaments from clustered sites of DNA damage (Fig. 3b). These DNA damage-induced intranuclear microtubule filaments (DIMs) contribute to DNA repair by increasing the mobilization of damaged chromatin within the nucleus^13,90^. The future identification of factors that may be enriched or excluded from such nuclear Rad52 condensates could provide additional unique insights into the relationship between LLPS and DNA repair.

## Conclusions and future directions

The growing impact of phase separation across cell and molecular biology has challenged our understanding of intracellular organization. The physicochemical properties of LLPS that underlie the assembly of various biomolecular condensates allow for a wide range of possible functions. Indeed, LLPS can efficiently organize the nucleus into distinct and specialized subcompartments that greatly differ in size and composition. These various condensates can serve to filter for specific biomolecular interactions that can either activate or restrict different biomolecular processes. LLPS is also emerging as a powerful mechanism in the control of biochemical reaction chains. Specifically, this is achieved by segregating, in space and/or time, the subsequent steps of a reaction or pathway into separate biomolecular condensates. These distinct condensates can be physically separated but still allowed to communicate with each other via the transfer of activated factors such as phosphorylated RNA Pol II from one condensate to the other^58,74^. However, distinct biomolecular condensates may independently assemble and disassemble at specific genomic locations after performing certain functions, such as DNA damage repair^12^.

The recent advances in the field of LLPS also raise several questions regarding the precise regulation and interplay of distinct biomolecular condensates inside of the nucleus. For example, how are the phase separation properties of heterochromatin affected by DNA damage in this compartment? One possibility is that pre-existing phase-separated compartments (such as an HP1-driven heterochromatin compartment) at sites of DNA damage might first need to disassemble to allow access to DNA repair factors and their organization via LLPS de novo. Consistent with this possibility, the assembly of biomolecular condensates can produce mechanical forces that are capable of dynamically restructuring the genome^91^. Such forces, combined with the action of chromatin remodelling enzymes, could enable the formation of low-chromatin density areas that favour the recruitment of DNA repair factors. It will be important to determine whether such mechanical reorganizations occur within heterochromatin compartments upon DNA damage. Alternatively, the mobilization of damaged DNA sites away from pre-existing phase-separated compartments might be required before the de novo assembly of biomolecular condensates favouring genome repair at the site of damage. In fact, the relocation of damaged DNA outside of repair-repressive nuclear domains does assist homologous recombination factors in gaining access to damaged DNA^92–94^. Moreover, in *S. cerevisiae*, Rad52 liquid-like droplets are assembled at damaged DNA and then transported onto microtubule filaments within the nucleus to promote repair^13^. Thus, future research should further explore the interplay between LLPS, damaged DNA movement and repair in different organisms^95^. In-line with these ideas, do the differences in phase separation properties between heterochromatin and euchromatin enable the specific recruitment of repair proteins at DNA damage sites? If different condensates constitute different “chromatin niches” for different DNA repair factors, this may also contribute to or even dictate DNA repair pathway choices and the level of activation of DNA damage checkpoint signalling. More globally, what factors are specifically enriched within or excluded from various biomolecular condensates? The identification of these factors through large-scale proteomics or imaging approaches will enable researchers to better appreciate the complexity and roles of these structures. For example, the identification of P body components paved the way to a better understanding of their regulation and functions in cells^96^. Finally, can we develop tools to allow us to study the features of individual biomolecular condensates in vivo? Thus, despite the immense global effort to understand the life cycles and functions of biomolecular condensates, when we consider the state of our knowledge of LLPS, it is clear that we still have many more questions than answers. Future studies that aim to answer these questions will undoubtedly provide unique insights into the intricate inner workings of the cell and may shed light on numerous human diseases.
